# [Corrigendum] Early anticoagulation therapy for severe burns complicated by inhalation injury in a rabbit model 

**DOI:** 10.3892/mmr.2025.13729

**Published:** 2025-10-29

**Authors:** Zhong-Hua Fu, Guang-Hua Guo, Zhen-Fang Xiong, Xincheng Liao, Ming-Zhuo Liu, Jinhua Luo

Mol Med Rep 16: 7375–7381, 2017; DOI: 10.3892/mmr.2017.7537

Following the publication of this paper, it was drawn to the Editor's attention by a concerned reader that, regarding the histological images shown in Fig. 1 on p. 7377, the ‘Sham’ and the ‘Model’ data panels appeared to show areas of overlapping data with the ‘Union’ and the ‘Antithrombin’ panels respectively, albeit with very different zoom factors, such that data which were intended to show the results from differently performed experiments had apparently been derived from the same original sources.

The authors were contacted by the Editorial Office to offer an explanation for these apparent anomalies in the presentation of the data in this paper, although, due to the time that had elapsed since our contacting them, an Expression of Concern (doi.org/10.3892/mmr.2025.13691) was issued as the Editorial Office continued to investigate this matter further. However, the authors, who still had access to their original data, have now responded to the query, and realize that the figure was inadvertently assembled incorrectly. The revised version of Fig. 1, now showing the correct data panels for the ‘Sham’, ‘Antithrombin’ and ‘Union’ panels, is shown below. Note that these errors did not significantly affect either the results or the overall conclusions reported in this study. All the authors agree with the publication of this corrigendum, and are grateful to the Editor of *Molecular Medicine Reports* for allowing them the opportunity to publish this. They also wish to apologize to the readership of the Journal for any inconvenience caused.

## Figures and Tables

**Figure 1.**
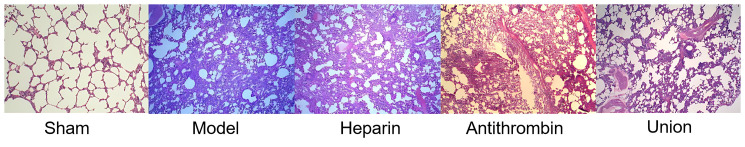
Effect of early anticoagulation therapy on lung injury in a rabbit model of severe burns complicated by inhalation injury. Magnification, ×40. Sham, sham group; model, severe burns model group; heparin, early heparin treatment group; antithrombin, early antithrombin group; union, early heparin + antithrombin group..

